# mSphere of Influence: a Systematic Approach To Dissect Virulence Traits in Candida albicans

**DOI:** 10.1128/mSphere.00258-19

**Published:** 2019-06-19

**Authors:** J. Christian Pérez

**Affiliations:** a Interdisciplinary Center for Clinical Research, University Hospital Würzburg, Würzburg, Germany; b Institute for Molecular Infection Biology, University Würzburg, Würzburg, Germany

**Keywords:** *Candida albicans*

## Abstract

J. Christian Pérez studies the interplay between Candida albicans and the mammalian host. In this mSphere of Influence article, he reflects on how “Systematic screens of a Candida albicans homozygous deletion library decouple morphogenetic switching and pathogenicity” (S. M. Noble, S. French, L. A. Kohn, V. Chen, et al., Nat Genet 42:590–598, 2010, https://doi.org/10.1038/ng.605) provided tools and a blueprint for open-ended genetic screens in an organism that had been a challenge for genetic manipulation.

## Abstract (precis)

J. Christian Pérez studies the interplay between Candida albicans and the mammalian host. In this mSphere of Influence article, he reflects on how “Systematic screens of a Candida albicans homozygous deletion library decouple morphogenetic switching and pathogenicity” (S. M. Noble, S. French, L. A. Kohn, V.

## COMMENTARY

In “Systematic screens of a Candida albicans homozygous deletion library decouple morphogenetic switching and pathogenicity,” Noble et al. (1) sought to identify novel C. albicans virulence determinants in an unbiased and systematic manner. The strategy employed in this endeavor was to carry out a genetic screen in a mouse model of disseminated infection. In addition to generating a large collection of fully vetted homozygous deletion strains—which became a resource that has been extremely valuable for the *Candida* research community to this day—Noble et al. demonstrated the feasibility and usefulness of interrogating a large pool of C. albicans genes directly in an animal system.

Despite its medical importance and inherent interest to researchers, progress in understanding the biology of C. albicans was somewhat slow compared to other microorganisms. This was due, in part, to the fact that the organism is diploid and yet lacks a complete sexual cycle, rendering conventional genetic analysis unfeasible. The construction of a substantial number of homozygous deletion strains by Noble et al. was arguably a milestone in the field because it facilitated the searches for fungal genes involved in traits of interest. Two major findings came from the initial genetic screen that Noble et al. performed in the standard tail vein murine infection model. First, they observed that a significant fraction of C. albicans genes with clear phenotypes in mice did not appear to influence the yeast-to-filament transition in this fungus. This was important because, until that time, the vast majority of “pathogenesis” genes seemed also linked in some manner to the ability of *Candida* to change its morphology. A second finding of the paper was the establishment of a glycosylceramide biosynthetic pathway as a virulence effector. This pathway played a role in the dissemination of the fungus to internal organs, but it did so without any major effect on the yeast-to-filament switch.

I became aware of the work of Noble et al. as a postdoc when I was starting to do C. albicans research (my doctoral work had been in bacterial genetics). The article published by Noble et al. was influential to me in two ways. First, it made me realize that this approach could represent a good entry point to investigate other aspects of the biology of C. albicans in the mammalian host. As a newcomer to the field, I had read that this fungus lives almost exclusively associated with a host (i.e., it has no known environmental reservoir) and yet only occasionally causes disease. Therefore, I was interested in exploring the broader host-*Candida* relationship, not only the “pathogen” facet. Indeed, the tools and experimental strategy described by Noble et al. provided a foundation to develop my own research projects on *Candida* commensalism (2, 3). A second way in which this publication influenced the direction of my research was that it made me more inclined to embrace the unpredictability of “open-ended” genetic screens. For my doctoral work, I had chosen to study a particular gene regulatory circuit that was already well known to contribute to key aspects of the host-microbe interplay. As a postdoc, however, I was eager to adopt a more global or genome-wide perspective that would allow me to identify (and then study) “novel” *Candida* gene circuits that shape the interactions between the fungus and its host. At that time, within the *Candida* field, the paper by Noble et al. was one of a handful (4, 5) that epitomized how some interesting and unexpected biological insights could be gained by having a wide net as a starting point.
